# More Quackery

**Published:** 1891-11

**Authors:** Sam. I. Fox

**Affiliations:** Willis, Texas


### MORE QUACKERY.

WILLIS, TEXAS, November 6, 1891.

*Editor Daniel's Texas Medical Journal:*

DEAR SIR—I send you report of case for publication :

About sixteen months ago, I was called to treat a colored woman for some trivial complaint. After a few days I pronounced her pregnant. In a few days I was discharged from the case, and it was placed in the hands of a different physician, who has been treating her for eight or ten months past for cancer of the womb, and has said all the time she would die. On Tuesday, November 3rd, in the absence of her regular physician, I was called to see her, in great haste. On reaching the bedside and making an examination, I found a partly decayed fœtus protruding from the *rectum*. After putting her under chloroform, I extracted it from the rectum. On examination I thought it was about three and a half or four months old when it died. I saw the woman on Thursday, and she seemed to be a great deal better, and I now think she will get well. Please publish in next JOURNAL. Yours respectfully, SAM. I. FOX.

